# Modulation of liver steatosis by miR-21/PPAR**α**

**DOI:** 10.1038/s41420-018-0076-z

**Published:** 2018-07-10

**Authors:** Pedro M. Rodrigues, Cecília M. P. Rodrigues, Rui E. Castro

**Affiliations:** Research Institute for Medicines (iMed.ULisboa), Faculty of Pharmacy, Universidade de Lisboa, Lisbon, Portugal

**Keywords:** Non-alcoholic fatty liver disease, Experimental models of disease

Non-alcoholic fatty liver disease (NAFLD) encompasses a spectrum of liver lesions ranging from simple steatosis to non-alcoholic steatohepatitis (NASH), eventually progressing to cirrhosis and hepatocellular carcinoma (HCC). Recent evidence indicates that microRNAs (miRNAs) and nuclear receptors are important players in disease progression, while also embodying promising therapeutic targets. Recently^1^, we showed that both liver and muscle miR-21 are increased in NASH patients and diseased mice, resulting in diminished expression of nuclear receptor peroxisome proliferator-activated receptor α (PPARα). Strikingly, miR-21 knockout (KO) mice fed a methionine-deficient and choline-deficient (MCD) diet display markedly reduced inflammation and fibrosis, alongside improvement of steatosis, when compared with wild type animals. Similar findings were previously reported by Loyer et al.^2^, except for improvements on lipid accumulation, as highlighted by Mazzini^3^. This discrepancy might result from singularities of each animal model. We^1^ used 5-month-old mice fed the MCD diet for 8 weeks, while Loyer et al.^2^ used 2-month-old mice and fed them for 10 weeks. Considering that the MCD diet induces quick disease progression, with development of NASH and fibrosis in a very short period of time, it may be that the effects of miR-21 ablation in steatosis are less evident after 10 weeks in younger animals. Nonetheless, the MCD model fails to mimic the metabolic profile found in NAFLD patients, with mice experiencing severe weight loss, low insulin, leptin and triglyceride serum levels and absent insulin resistance; not the most suitable model to study lipid metabolism. Consequently, miR-21 function has also been evaluated in the fast food (FF) NASH mouse model, mimicking most metabolic features of human patients. In this scenario, we found that miR-21 ablation resulted in a slight reduction in steatosis, which was more pronounced when combined with the farnesoid x receptor (FXR) agonist obeticholic acid (OCA). In fact, this prospective therapeutic approach restored hepatic lipid metabolism, decreasing fatty acid uptake and re-establishing β-oxidation to basal levels, ultimately reducing steatosis and cholesterol accumulation. Calo et al.^4^ recently reported similar results, where miR-21 KO mice fed a high fat diet (HFD) exhibited significantly decreased hepatic steatosis, corroborating that miR-21 ablation impacts on hepatic lipid metabolism. Increased O_2_ consumption, as well as heat and CO_2_ production was further observed, suggesting enhanced energy expenditure in miR-21 KO mice. Altogether, results from different obesogenic models suggest that miR-21 ablation might, indeed, impact on hepatic steatosis.

**Fig. 1:**
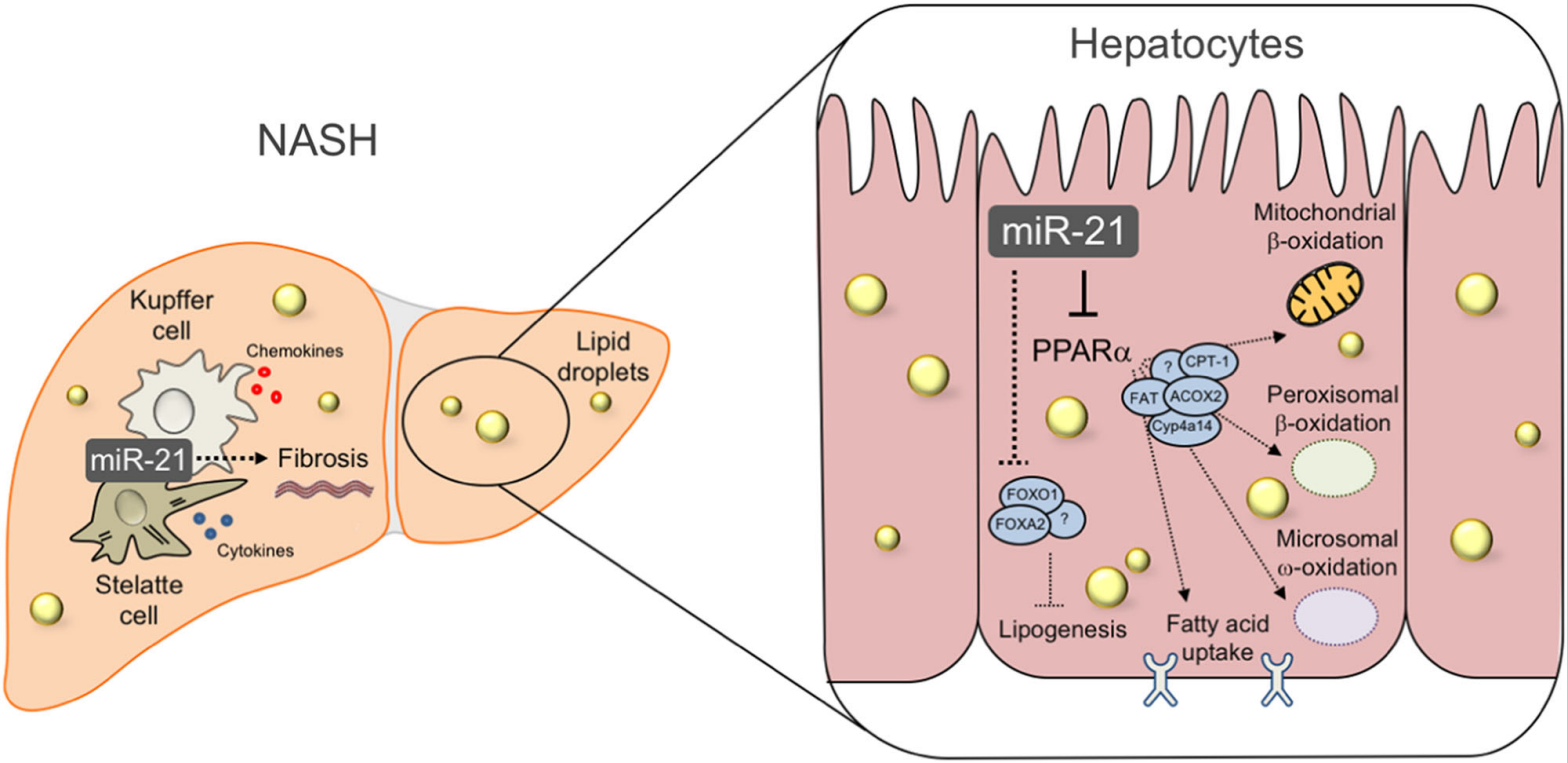
Effects of liver miR-21 in NAFLD pathogenesis During NASH, liver miR-21 is primarily expressed in inflammatory (and biliary) cells, contributing to overall cellular injury, inflammation and fibrosis, mostly through PPARα inhibition (left). Recent findings suggest that hepatocyte miR-21 also plays a role in development of steatosis, trough inhibition of PPARα-mediated lipid oxidation and fatty acid uptake, as well as through PPARα-independent modulation of genes involved in lipogenesis (right). ACOX2, Acyl-CoA Oxidase 2; CPT-1, Carnitine palmitoyltransferase I; Cyp4a14, cytochrome P450 4A14; FAT, fatty acid translocase; FOXA2, Forkhead box A2; FOXO1, Forkhead box O1

Loyer et al. also reported that miR-21 expression was predominantly found increased in biliary (CK19^+^) and inflammatory cells (CD3^+^), rather than in hepatocytes, of both mice and NASH patients. As such, it would be fair to hypothesize that the effects of inhibiting miR-21 might greatly impact these cells while having little effects in hepatocytes and/or steatosis. However, the phenotype of hepatocyte-specific miR-21 KO mice (LImiR21KO) is very similar to that of whole-body mir-21 KO mice^4^; LImiR21KO challenged with a HFD for 4 weeks displayed increased glucose tolerance and increased hepatic insulin sensitivity. Moreover, these mice exhibited a higher ratio of lean versus fat body mass and markedly reduced hepatic steatosis, paralleling decreased expression of genes involved in fatty acid uptake and lipogenesis, including fatty acid synthase, CD36 and FATP1. Similarly, we report^1^ that miR-21 KO mice display reduced hepatic expression levels of genes involved in fatty acid transport and in lipogenesis (Fig. 1). Of note, miR-21 inhibition was also shown to ameliorate alport nephropathy in mice through stimulation of different metabolic pathways^5^.

PPARα is a target of miR-21 during NAFLD pathogenesis^6^. In fact, PPARα masters lipid β-oxidation in hepatocytes and exerts anti-inflammatory effects in non-parenchymal cells. As such, the improved phenotype of miR-21 KO mice might result, at least in part, from the pro-metabolic and anti-inflammatory effects of PPARα in different liver cells. To further confirm the role of PPARα in lipid metabolism, we also measured the expression of direct transcriptional metabolic relevant targets of PPARα that were shown to be significantly increased in miR-21 KO mice. Of note, the anti-inflammatory effects of PPARα might also positively impact on liver steatosis^7^. Nonetheless, we agree with Mazzini in that the role of the miR-21/PPARα axis, particularly in hepatocytes, does deserve further studies.

The network in which miR-21 in involved is likely much more complex than it would initially appear. PPARα is surely not the sole direct target of miR-21 being regulated in NASH. For example, Calo et al. also reported that the anti-steatogenic effects of knocking out miR-21 in hepatocytes alone could relate with subsequent increased expressions of FOXO1, FOXA2, and HNF4α (Fig. 1). Moreover, deletion of PTEN, another miR-21 target, associates with massive hepatomegaly, triglyceride accumulation, inflammation and spontaneous HCC in mice^8^. A more direct role of miR-21 in liver fibrosis could also be envisaged; miR-21 has been shown to activate HSCs, while its inhibition robustly decreases liver fibrosis through induction of CD24+ cell-dependent apoptosis^9^. miR-21 may also exert its pathological effect in NAFLD by acting upon extrahepatic tissues. Kim et al. reported that human adipose tissue-derived mesenchymal stem cell proliferation is modulated by miR-21, found deregulated in white adipose tissue of HFD-fed mice^10^. In addition, long-term inhibition of miR-21 was shown to ameliorate obesity in *ob*/*ob* mice^11^, highlighting the potential of miR-21 anti-sense therapies in metabolic diseases, including NAFLD. Finally, the HBP1/p53/SREBP-1c pathway possible links miR-21 to the progression of NASH towards HCC^12^. Altogether, it appears that miR-21 inhibition ameliorates NAFLD progression through a complex network of events. Considering that nuclear receptor-targeted therapies are still unsatisfactory, its combination with anti-miR-21 strategies might prove incrementally successful.
